# Construction of a recombinant avipoxvirus expressing the *env* gene of Zika virus as a novel putative preventive vaccine

**DOI:** 10.1186/s12985-021-01519-x

**Published:** 2021-03-04

**Authors:** Carlo Zanotto, Francesca Paolini, Antonia Radaelli, Carlo De Giuli Morghen

**Affiliations:** 1 Laboratory of Molecular Virology and Recombinant Vaccine Development, Department of Medical Biotechnologies and Translational Medicine, University of Milan, Via Vanvitelli 32, 20129 Milan, Italy; 2 HPV-UNIT, Laboratory of Virology, Regina Elena National Cancer Institute, Via delle Messi d’Oro, 156, 00158 Rome, Italy; 3 Catholic University “Our Lady of Good Counsel”, Rr. Dritan Hoxha, 123, Tirana, Albania

**Keywords:** Zika virus, Recombinant vaccines, Fowlpox virus, Prime–boost vaccination, Immune response, Electron microscopy

## Abstract

**Background:**

Zika virus (ZIKV) has been declared a public health emergency that requires development of an effective vaccine, as it might represent an international threat.

**Methods:**

Here, two novel DNA-based (pVAX*zenv*) and fowlpox-based (FP*zenv*) recombinant putative vaccine candidates were constructed that contained the *cPrME* genes of ZIKV. The *env* gene inserted into the fowlpox vector was verified for correct transgene expression by Western blotting and by immunofluorescence in different cell lines. The production of virus-like particles as a result of *env* gene expression was also demonstrated by electron microscopy. BALB/c mice were immunosuppressed with dexamethasone and immunized following a prime–boost strategy in a heterologous protocol where pVAX*zenv* was followed by FP*zenv*, to evaluate the immunogenicity of the Env protein. The mice underwent a challenge with an epidemic ZIKV after the last boost.

**Results:**

These data show that the ZIKV Env protein was correctly expressed in both normal human lung fibroblasts (MRC-5 cells) and green monkey kidney (Vero) cells infected with FP*zenv*, and that the transgene expression lasted for more than 2 weeks. After mucosal administration of FP*zenv*, the immunized mice showed specific and significantly higher humoral responses compared to the control mice. However, virus neutralizing antibodies were not detected using plaque reduction assays.

**Conclusions:**

Although BALB/c mice appear to be an adequate model for ZIKV infection, as it mimics the natural mild infection in human beings, inadequate immune suppression seemed to occur by dexamethasone and different immune suppression strategies should be applied before challenge to reveal any protection of the mice.

## Highlights

1. A recombinant avipoxvirus was constructed to express the *env* gene of Zika virus
2. Novel putative recombinant vaccines were used in a prime–boost immunization regimen
3. Mucosal immunization enhances the humoral immune response

## Background

Zika virus (ZIKV) was first isolated in 1947 from rhesus macaques in the Zika Forest Research Station of Uganda, and was then identified in *Aedes africanus* mosquitoes from the same forest [1]. ZIKV belongs to the Flavivirus genus of the *Flaviviridae* family, which includes the Dengue, Yellow fever, West Nile, Japanese encephalitis, and Tick-borne encephalitis viruses, which have single-stranded, positive-sense RNA genomes of around 11 kb [2]. In particular, ZIKV shows antibody cross-reactivity to the four serotypes of Dengue virus [3]. Although ZIKV might also be sexually and vertically transmitted [4, 5], bites by *Aedes aegypti* and *Aedes albopictus* mosquitos represent the main route of ZIKV infection in humans [6].

Human infections were initially reported in Nigeria in 1954 [7], but the first major outbreak occurred in 2007 on Yap Island, in the Federated States of Micronesia, where almost 75% of the population was shown to be infected, and almost 20% developed symptomatic disease [8]. Large outbreaks also occurred in French Polynesia in 2013 [9], and in South America [10]. ZIKV infections are mainly asymptomatic, but in spite of the generally mild self-limiting symptoms associated with maculopapular rash, headache, conjunctivitis, and musculoskeletal pain, neurological complications can occur, such as microcephaly in the developing fetus [4]. ZIKV has also been associated with Guillain-Barré syndrome in adults, an autoimmune neurological disease that is characterized by muscle weakness, motor dysfunction, and in some cases, paralysis [10, 11], as the virus can infect human neural progenitor cells [12].

Thus, since its introduction into Brazil in 2015, ZIKV has been declared a public health emergency of international concern by the World Health Organization [13], as it might represent an international threat [14]. Considering also its easy transmission from asymptomatic patients, rapid development of a safe and effective vaccine is required to prevent further outbreaks.

Currently, there have been many attempts to develop candidate vaccines against ZIKV [15–17] that have included subunit and recombinant plasmid-based vaccines, inactivated or live-attenuated viral vaccines, recombinant vaccines [18, 19], and virus like particles (VLPs) [20, 21]. All these have shown different efficacies in mice and nonhuman primate models [20, 22–27]. Some of them have also been advanced to clinical evaluation, and are undergoing phase I and II clinical trials [12, 13, 28, 29].

Attenuated viral-vectored vaccines are among the most effective immunogens against infectious diseases [30, 31], as they are potent stimulators of antibodies and cell-mediated immunity, and they can protect against both homologous and heterologous virus strains [32]. In particular, avipox viruses have taken on an important role in the development of novel recombinant immunogens, as they do not replicate in most mammalian cells, although permissive for entry and transgene expression [33, 34]. Moreover, avipoxvirus vectors do not cause the undesired side effects induced by vaccinia-based recombinants, and they are not neutralized in individuals who have already been immunized against smallpox [35]. In particular, Fowlpox (FP)-based recombinants can express foreign antigens for long periods and induce protective immunity in mammals [36–38].

The structural proteins encoded by ZIKV after post-translational processing of the RNA genome include the capsid (c), the membrane precursor or pre-membrane (Pr), the membrane (M), and the envelope (E) proteins. In particular, the envelope proteins of flaviviruses show very similar structures and functions, as they can mediate virus cell fusion [39] and elicit a cell-mediated response [40]. They are therefore the main targets of neutralizing antibodies, and can be related to ZIKV neurotropism [39].

**Fig. 1:**
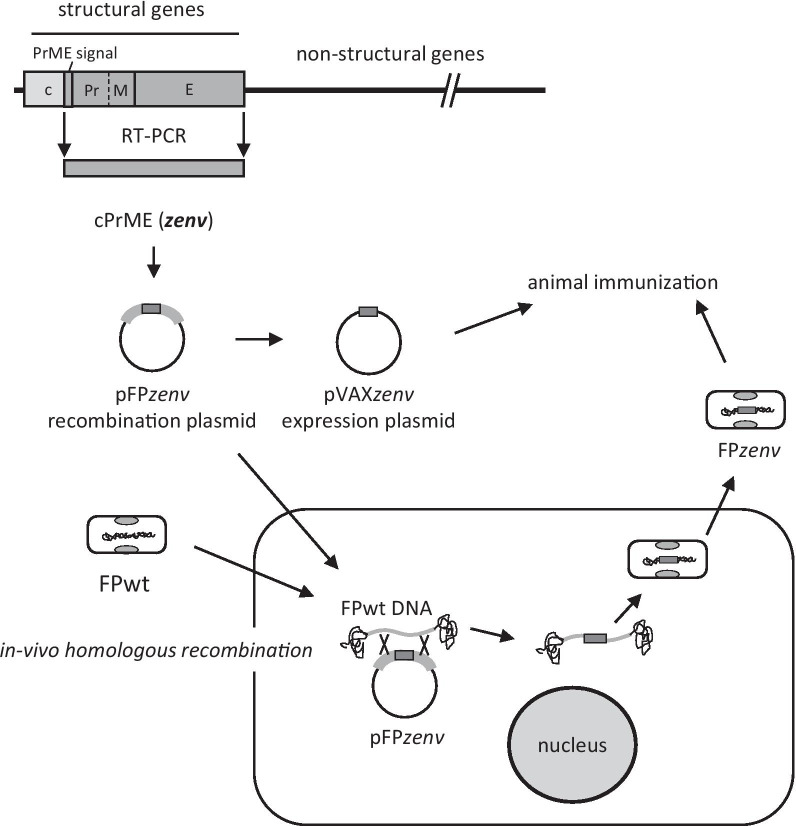
Plasmids construction and *in-vivo* recombination. Two plasmids were constructed: pFP*zenv* as the recombination plasmid for *in-vivo* recombination and pVAX*zenv* as the expression plasmid to prime the experimental mice. pFP*zenv* contains the *cPrME* gene sequence of ZIKV (*zenv*) obtained by retro-transcription of a 2015 Brazilian isolate. This sequence includes the genes that encode for the tail portion of the capsid protein (c), the membrane precursor (Pr), the membrane protein (M) and the whole envelope protein (E). Homologous *in-vivo* recombination occurred in specific pathogen-free primary chick embryo cells after infection of FPwt and transfection of the pFP*zenv* recombination plasmid. Recombinant plaques were identified by autoradiography after hybridization with the [^32^P]-labelled *zenv* probe and different positive clones were subjected to multiple cycles of plaque purification until one clone (FP*zenv*) was selected for correct expression

Here, we report on the construction of a novel DNA recombinant (pVAX*zenv*) and a novel FP recombinant (FP*zenv*) putative vaccines that contain the *cPrME* genes of ZIKV (Fig. 1). This sequence is related to cellular entry, and we evaluate the immunogenicity of the Env protein in a mouse model after challenge with an epidemic ZIKV strain. The final aim was to use the pVAX*zenv* recombinant as a prime and the FP*zenv* recombinant as a boost, administered also by the mucosal route. This novel FP construct was used to infect chick embryo fibroblasts (CEFs), normal human lung fibroblasts (MRC-5 cells), and green monkey kidney (Vero) cells to assess transgene expression in vitro. Transcript expression in Vero cells was tested to determine whether FP*zenv* induces long-lasting responses. The production of VLPs, as a result of *env* gene expression, was also verified by electron microscopy. Mice immunization was performed by priming the animals with pVAX*zenv* by *in-vivo* electroporation (e.p.) and boosting them by subcutaneous (s.c.) and intranasal (i.n.) administration of the FP*zenv*. Humoral responses were verified before all bleeding times, and the virus neutralizing activity was tested before the challenge. The challenge with ZIKV was performed, after the last boost, on mice immunosuppressed with dexamethasone. The experimentally immunized mice showed significantly higher antibody responses compared to the controls, especially after FP*zenv* administration by the mucosal route. Viral neutralizing activity could not be demonstrated, as well as protection after the challenge with ZIKV, as all of the mice survived.

## Material and methods

### Cells

Specific-pathogen-free primary CEFs were grown in Dulbecco’s Modified Eagle’s Medium (DMEM) supplemented with 5% heat-inactivated calf serum (Gibco Life Technologies, Grand Island, NY, USA), 5% Tryptose Phosphate Broth (Difco Laboratories, Detroit, MI, USA), 100 U/mL penicillin, and 100 µg/mL streptomycin. MRC-5 and Vero cells were grown in DMEM supplemented with 10% heat-inactivated calf serum, with 100 U/mL penicillin and 100 µg/mL streptomycin.

### Recombination plasmid

The plasmid prepared for *in-vivo* recombination contained the *cPrME* gene sequence of ZIKV (Fig. 1). This fragment included the whole envelope gene sequence (54.38 kDa; 504 amino acids), as well as the genes that encode for part of the capsid protein (~ 2 kDa; 18 amino acids), the membrane precursor (10.12 kDa; 92 amino acids), and the membrane protein (8.4 kDa; 76 amino acids) [41]. In particular, the capsid hydrophobic tail is a signal peptide for the translocation of PrM to the endoplasmic reticulum [42], whereas PrM prevents the rearrangements of the envelope proteins in an acidic milieu, and their fusion with cell membranes during translocation through the secretory pathway.

The ZIKV RNA genome was obtained from the serum of a 2015 Brazilian patient (ZikaSPH2015 strain) and was supplied by the EVAg project through the courtesy of M.R. Capobianchi (National Institute for Infectious Diseases L. Spallanzani, I.N.M.I., Rome, Italy). It was retro-transcribed and amplified using the forward V436 (5′ CCG CGC CCG GGA AGC TTA TGG GCG CAG ATA CTA GTG TC 3′) primer and reverse V437 (5′ GGG GTA CCG CGG CCG CAT AAA AAT TAA GCA GAG ACG GCT GTG GA 3′) primer, to get the *cPrME* fragment. The primers were designed to include the SmaI/HindIII sites at the 5′ end, followed by the ATG sequence, and the NotI/KpnI sites at the 3′ end. These sites were needed to clone the ZIKV *cPrME* gene fragment into the pFP recombination plasmid. At the 3′ end, a T5NT sequence was also added, as an additional poxviral transcription termination signal.

RNA (10 ng) was retro-transcribed using Transcriptor One-Step RT-PCR kit (Roche Molecular Systems, Indianapolis, IN, USA) in 50 μL following the manufacturer instructions.

After deletion of the *A27L* gene from pFP_A27L_ recombination plasmid [43, 44] and subcloning of the *cPrME* fragment, the resulting pFP*cPrME*18 clone was sequenced to exclude any possible mistakes due to the PCR amplification. A non-synonymous mutation at nucleotide 759, where the cytosine nucleotide had replaced the thymine giving origin to an alanine instead of a valine, was corrected by site-specific mutagenesis [45, 46].

The *cPrME* mutagenized fragment was thus inserted inside the 3-β-hydroxysteroid dehydrogenase 5-delta 4 isomerase gene, downstream of the Vaccinia virus H6 (H6) early/ late promoter [47]. The sequence was aligned with the *env* gene of ZIKV (GenBank accession number KU991811) using Align Plus 2.0. This pFP*cPrME* recombination plasmid (10,274 bp) was finally designated as pFP*zenv*.

### Recombinant fowlpox virus

The FP*zenv* viral recombinant putative vaccine was generated by *in-vivo* homologous recombination [48]. Briefly, FP*zenv* was obtained on specific pathogen-free primary CEFs, using the recombination plasmid pFP*zenv* described above (62.5 µg) and the wild-type FP virus (5 PFU/cell). Recombinant plaques were identified by autoradiography after hybridization with the [^32^P]-labelled *zenv* probe. Recombinants were subjected to multiple cycles of plaque purification until one clone was selected for correct expression. The recombinant was amplified in CEFs, purified on discontinuous sucrose density gradients, and titrated essentially as described previously [49]. Briefly, the cells were harvested, ultracentrifuged at 30,000 × *g* for 2 h at 4 °C, and the pellets were resuspended in 1 mM Tris, 150 mM NaCl, 1 mM EDTA, pH 7.4. After addition of trypsin (0.06% final concentration), the pellet was incubated for 5 min at 37 °C, and the virus was released from the cells by sonication. The supernatant was overlaid onto a discontinuous 30% to 45% (w/w) sucrose gradient, in the same buffer. After ultracentrifugation at 38,000 × *g* for 1 h, the viral band at the interface was recovered, diluted with 1 mM Tris–HCl, pH 9, and pelleted at 67,000 × *g* for 1 h. The purified virus was resuspended in Ca^2+^-free and Mg^2+^-free phosphate-buffered saline (PBS^–^), briefly sonicated, and then aliquoted and frozen at − 80 °C until use.

### Expression plasmids

Two expression plasmids, pVAX*gag/proM766* (here referred to as pVAX*gp*) and pVAX*zenv*, were used to prime the mice. pVAX*gp* contains the SIVmacM766 *gag/pro* gene [50], which was a kind gift from G. Franchini (National Cancer Institute, NIH, Bethesda, MD, USA), and was used as an irrelevant negative control. The *zenv* gene was excised from pFP*zenv* and inserted into the pVAX expression plasmid (Invitrogen Corp., San Diego, CA, USA), which contained the human CMV promoter and is approved for use in humans. Transformation was performed using JM109 competent bacteria, in the presence of 50 µg/mL kanamicin, as pVAX contains the kanamicin resistance gene. Briefly, the *zenv* gene was cut from pFP*zenv* with HindIII/NotI and inserted into the pVAX*envM766* plasmid, from where the *envM766* gene had been previously removed using the HindIII/NotI/SalI restriction enzymes. Bacterial selection was performed by PCR amplification using the V438/V441 primers, and 2.5 mM MgCl_2_. Amplification was carried out starting from 1 μL of each bacterial colony in a final volume of 20 μL in a mixture containing 1 μM of each primer, 200 μM of each dNTP, 2.5 mM MgCl_2_, and 0.025 U/µL Taq DNA polymerase (Fermentas). The PCR conditions were: 94 °C for 2 min, followed by 30 cycles at 94 °C for 30 s, 51 °C for 30 s, 72 °C for 45 s, and extension at 72 °C for 7 min (PTC-200 thermocycler; MJ Research, Waltham, MA, USA).

### ZIKV amplification

Vero cells were infected for 1 h at 37 °C with 0.4 PFU ZIKV (MR766 strain), a kind gift from M.R. Capobianchi (I.N.M.I.), and maintained in DMEM with 5% heat-inactivated calf serum. After 5 days, the cells were harvested, disrupted, and centrifuged at 400 × *g* for 5 min. The supernatant was then aliquoted and titered according to the Vero cells.

### Western blotting

To determine whether the Env protein was expressed, replication nonpermissive Vero cells were infected for 1 h at 37 °C using FP*zenv* (10 PFU/cell). After overnight incubations, the samples were collected, run on 12.5% polyacrilamide gels, and examined by Western blotting, as described previously [51, 52]. The blotted nitrocellulose membranes were incubated overnight at 4 °C using the human polyclonal anti-ZIKV–specific serum (dilution, 1:200), and rabbit polyclonal antibodies or mouse monoclonal antibodies (always at dilution 1:500). The primary antibodies were followed by horseradish-peroxidase-conjugated secondary antibodies, as goat anti-human serum (dilution, 1:1,000; DakoCytomation, Carpinteria, CA, USA) or goat anti-rabbit (dilution, 1:2,000) or goat anti-mouse (dilution, 1:1,000). After a 1-h incubation and 2-h washes, the proteins were revealed using the ECL system (Western Lightning Plus-ECL; PerkinElmer, Waltham, MA, USA) followed by exposure of the nitrocellulose membranes to a hyperfilm for different times (Amersham Hyperfilm ECL; GE Healthcare, Buckinghamshire, UK). Cells infected with FP wild-type and with ZIKV were used as negative and positive controls, respectively.

### Immunofluorescence

Immunofluorescence was carried out as already described [53], using CEFs and Vero and MRC-5 cells, to examine the expression and subcellular localization of the ZIKV Env protein. Briefly, the cells were seeded at a density of 5 × 10^5^/35-mm-diameter dish on sterile glass coverslips. After infection with FP*zenv* (5 PFU/cell; except for CEFs, which were infected with 0.5 PFU) at 37 °C for 1 h, the cells were incubated overnight at 37 °C in DMEM supplemented with 2% fetal calf serum. The cells were then washed twice with PBS^–^, and fixed with 2% paraformaldehyde (Polysciences) in PBS^–^ for 10 min at room temperature, followed by 100% cold acetone for 5 min at 4 °C. The samples were incubated with the 1:100-diluted human polyclonal anti-ZIKV serum, which was a kind gift from M.R. Capobianchi, or with the 1:50-diluted rabbit polyclonal anti-Env serum (GeneTex Int. Corp., Inc., Irvine, CA, USA), or with the 1:50-diluted mouse monoclonal anti-Env antibody (GeneTex). The primary antibody was followed by the 1:50-diluted FITC goat anti-human or sheep anti-rabbit or goat anti-mouse antiserum (Cappel, MP Biomedicals, Inc., Aurora, OH, USA). FPwt and ZIKV that were previously produced in our laboratory were used to infect the cells, as negative and positive controls, respectively. The samples were viewed under a fluorescence microscope (Axioskop; Zeiss).

### Ultrastructural analysis by transmission electron microscopy

Confluent MRC-5 and Vero cells were infected with 6 or 4 or 2 or 1 PFU/cell FP*zenv*, and CEFs with 0.05 or 0.1 or 0.5 PFU/cell, for 1 h at 37 °C, and were collected 3 days post infection (p.i.). ZIKV was used at 1 PFU/cell as the positive control. Inclusion was performed as already described [38]. Briefly, after centrifugation at 1,000 × g for 10 min, the cells were all fixed in 2.5% glutaraldehyde (Polysciences, Warrington, PA, USA) in 0.1 M Na cacodylate buffer, pH 7.4, for 1 h at 4 °C, and then rinsed twice and post-fixed in cacodylate-buffered 1% OsO_4_ at 4 °C for 1 h. The specimens were dehydrated through a series of graded ethanol solutions and propylene oxide, and embedded in Poly/Bed 812 epoxy resin mixture. Sectioning was performed with an ultramicrotome (MT2B; Sorvall, New York, NY, USA) equipped with a diamond knife. After staining with water-saturated uranyl acetate and 0.4% lead citrate in 0.1 M NaOH, ultra-thin sections were examined using an electron microscope (CM10; Philips, Eindhoven, The Netherlands).

### Expression over time of viral RNA transcripts in Vero cells

Confluent replication-restrictive Vero cells (1.5 × 10^6^ cells/Petri dish; diameter, 5 cm) were infected with FP*zenv* at 5 PFU/cell for 1 h at 37 °C. The cells were rinsed twice with PBS^−^, scraped from the Petri dishes with a rubber policeman every 3 days for 4 weeks, and centrifuged at 1500 × *g* for 5 min at room temperature. Cell lysis and RNA extraction were performed according to the QIagen RNeasy mini kit protocol, following the manufacturer instructions, with minor modifications. Briefly, 350 µL RLT lysis buffer was added to the cell pellets, which were resuspended before freezing at − 80 °C. When all of the samples were ready, RNA extractions started by adding to each sample one volume 75% ethanol. The RNA was transferred to the kit columns, which were then centrifuged for 15 s at 8000 × *g* at room temperature. The columns were washed four times with wash buffer, as indicated by the manufacturer. The DNase treatment, after the first wash/ centrifugation cycle with 500 µL RPE, was also performed using the DNaseI incubation mix (QIagen, RNase-free DNase sets; 10 µL DNaseI in 70 µL RDD buffer). After the last wash with 500 µL RPE, elution was performed with 60 µL RNase/DNase-free water, and the RNA concentrations were determined using a spectrophotometer (SmartSpec 3000; BioRad, Hercules, CA, USA). RT-PCR was performed using RT-PCR system kit (Access; Promega, Madison, WI, USA). Briefly, 50 ng RNA was used in a final volume of 20 μL in the presence of 1 μM of each primer, 250 μM of each dNTP, 1 U *Thermus filiformis* DNA polymerase, 1 U Avian Myeloblastosis Virus reverse transcriptase, and 3 mM MgSO_4_. The ZIKV *env*-specific primers V438 and V441 were used to obtain a 661-bp fragment. RNAs from ZIKV-infected and noninfected Vero cells were used as positive and negative controls, respectively. The reverse transcriptase reaction was performed at 45 °C for 45 min, followed by 2 min at 94 °C. PCR amplification was carried out for 40 cycles at 94 °C for 30 s, 58 °C for 30 s, and 68 °C for 45 s, followed by a final incubation at 68 °C for 7 min. β-actin was amplified, which gave a band of 518 bp using 5 ng RNA in a final volume of 20 µL, under the conditions described above, except that 1 mM MgSO_4_ was used. Primers V84 (5′ CTG ACT ACC TCA TGA AGA TCC T 3′ nt 630–651) and V85 (5′ GCT GAT CCA CAT CTG CTG GAA 3′ nt 1147–1127) were used. The PCR products were run on 1% agarose gels, and gel images were acquired by Speedlight Platinum apparatus (Lightools Research, Encinitas, CA, USA).

### Immunization protocols

Two groups of 7-week-old female BALB/c mice were used (Charles River Laboratories, Wilmington, MA, USA), as seven mice/group (Fig. 3**a**). For the control Group 1 (G1), we used the pVAX*gp* plasmid (10 + 50 µg/mouse), followed by FP*gp* (1 × 10^6^ PFU/mouse), where both the plasmid and the viral recombinants contain the same irrelevant SIVmacM766 *gag/pro* gene, previously described. For the experimental Group 2 (G2), we used the pVAX*zenv* plasmid (10 + 50 µg/mouse), followed by FP*zenv* (1 × 10^6^ PFU/mouse) where both the plasmid and the viral recombinants contain the same ZIKV *zenv* gene, previously described. Before each immunization, the mice were anesthetized by intramuscular (i.m.) injection of 30 µL of a mixture of 3.5 µL Rompun (stock, 20 mg/mL; Bayer SpA, Milan, Italy) plus 5.7 µL Zoletil 100 (Virbac Srl, Milan, Italy) and 35.7 µL PBS^–^. The vaccination course with pVAX*gp* or pVAX*zenv* consisted of 50 μg i.m. injection and 10 µg s.c. injection, followed by electroporation. For the electroporation, one 50-ms transcutaneous low-voltage electric pulse (amplitude, 100 V) was administered at the i.m. injection site via a multiple-needle electrode connected to the electroporation apparatus (ECM830, BTX i45-168, Holliston, MA, USA). Priming was followed by four boost administrations of FP*zenv*: two s.c., one i.n., and one both i.n. and s.c.. Challenge with ZIKV (1 × 10^5^ PFU/mouse) was performed s.c. at 10 days after the last immunization. For 6 days before the ZIKV challenge and for 4 days after the ZIKV challenge, the mice were immune suppressed with dexamethasone (Soldesam, 4 mg/mL; LFM, Milan, Italy) intraperitoneally [54]: 50 mg/kg on the first 2 days, and 25 mg/kg for the following days. Bleedings were performed from the retro-orbital eye plexus before the first immunization (Fig. 3**a**, T0), before each subsequent immunization (Fig. 3**a**, T1–T5), and at different intervals thereafter, as indicated. The plasma fractions were aliquoted and frozen at − 80 °C. Dexamethasone was withdrawn 4 days after the challenge (T9) and 4 days before the sacrifice (T10, T11).

The mice were also monitored during the whole treatment period for weight loss until euthanasia. The experimental group did not show any significantly differences in weight compared to the control mice, with the weight variations seen as < 15%, compared to the starting period. All of the mice were maintained according to the Italian National Guidelines and the EU Directive 2010/63/EU for animal experiments. They were observed for signs of disease, and provided with food and water ad libitum. Every effort was made to minimize their suffering. Approval for this study was granted by the Ethical Committee of the University of Milan.

### Enzyme-linked immunosorbent assay

The mouse plasma samples from T0 to T11 were assayed for antibodies against ZIKV Env-specific proteins using enzyme-linked immunosorbent assays (ELISAs). Vero cells (1.5 × 10^6^) previously infected for 2 days with FP*zenv* (2 PFU/cell) were used as the antigen, after plating in 96-well microtiter plates (MaxiSorp; Nunc, Thermoscientific, Roskilde, Denmark). Briefly, after infection and washing with PBS^–^, the cells were freeze-thawed three times, harvested with a rubber policeman, passed through the needle of an insulin syringe (30 G × 8 mm), and centrifuged for 5 min at 800 × *g*. Following resuspension in 0.05 M carbonate-bicarbonate buffer, pH 9.6 (15 mM Na_2_CO_3_, 35 mM NaHCO_3_, 0.2% NaN_3_), 1 × 10^5^ cells in 50 µL were added to the wells of 96-well plates. The antigen was incubated overnight at 4 °C. ELISAs were performed in duplicate, essentially as described previously [55], using serum from each animal of both groups of mice (G1, control group; G2, experimental group) from T0 to T11. The sera dilutions were 1:1,000. The reactions were revealed using goat anti-mouse horseradish-peroxidase-conjugated serum (dilution, 1:1,000; DakoCytomation, Glostrup, Denmark) and tetramethylbenzidine substrate (Sigma–Aldrich). The pre-immune mouse sera (T0) were used as the negative controls. The absorbance of each well was read at 450 nm using a microplate reader (550; Bio-Rad, Hercules, CA, USA). Inactivated ZIKV (4 × 10^5^ PFU/well) and the recombinant ZIKV Env-specific protein (10–300 ng, ZIKV envelope domain III, European Virus Archive goes Global, EVAg, Marseille, France) were also used as antigens (serum dilution, 1:100).

### Virus neutralization assays

The neutralizing activities of the mice sera were determined by measuring the extent of *in-vitro* inhibition of virus infectivity at T0 (pre-immune serum) and T6 (pre-challenge serum). The assays were performed as previously described [44], by pre-incubation of an equal volume of ZIKV with heat-inactivated mouse serum, used at different dilutions (1:50 to 1:1600, in DMEM without serum) in 48-well plates, for 1 h at 37 °C. Briefly, the viral titer was adjusted to provide approximately 80 PFU ZIKV in the assays. The infections were performed in duplicate on confluent Vero cells, and were allowed to proceed for 1 h at 37 °C. The same amount of virus incubated with DMEM was used as the control. Two days later, 5 mL medium was added to maintain the correct pH, and 5 days p.i. the cells were fixed in 3 mL methanol:acetic acid (3:1; v/v) for 1–3 h at room temperature. After removing the fixing solution and the agarose overlay, staining was performed using 1 mL 2% crystal violet dye in methanol. The neutralizing activity is expressed as the plaque reduction numbers and calculated by comparing the plaque numbers after incubating the virus with immune sera to the plaque numbers found after incubating the virus with no serum or with pre-immune sera.

### Viral RNA amplification to determine ZIKV after the challenge

To determine whether ZIKV was present after the challenge in the vaccinated mice, the viral RNA was extracted from the sera obtained at T7-T11 from the control and experimental mice, using QIAamp viral RNA mini kit (QIagen), according to the manufacturer instructions. Amplifications were performed using 50, 70, 250, 280, 400 ng of each RNA. Sera of some individual mice were also tested using 800 ng RNA. RT-PCR was performed using RT-PCR system kit (Access; Promega), as described above, using primers V438/V441 and under essentially the same conditions, with 3 mM MgSO_4_ and 58 °C annealing temperature, which was the most suitable for ZIKV detection.

### Statistical analyses

Statistical analyses were performed using parametric t-tests and areas under the curves (AUCs), using the GraphPad Prism version 2.0 software. Statistical significance was set as p < 0.05 (*), p < 0.01 (**) and p < 0.001 (***).

## Results

### Env is expressed by FP*zenv* in Vero cells

**Fig. 2:**
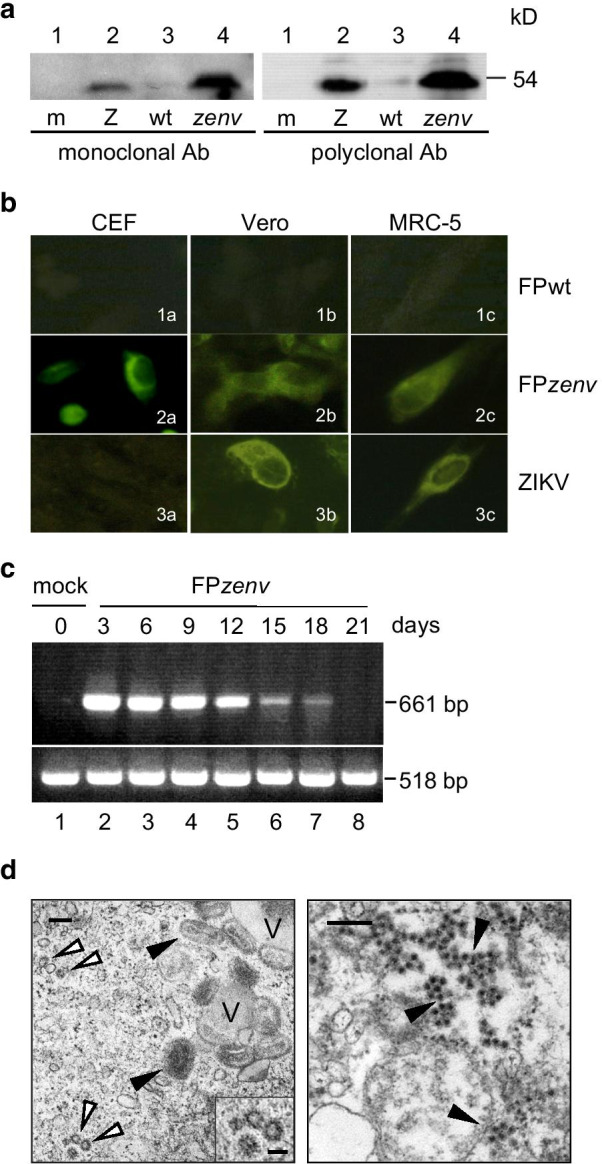
In vitro characterization of FP*zenv*-mediated transgene expression and VLP formation. **a** Expression of the Env protein of ZIKV by the FP recombinants in Vero cells. Vero cells were infected by the FP recombinants and examined using Western blotting, to determine the Env protein expression. The Env protein was always detected both when using the monoclonal or the polyclonal antibodies after infection with either ZIKV (Z, lanes 2) or FP*zenv* (*zenv*, lanes 4). Mock infected cells (m, lanes 1) and cells infected with FP wild-type (wt, lanes 3) were used as negative controls. **b** Heterologous protein expression by immunofluorescence in the CEFs and the Vero and MRC-5 cells. Immunofluorescence of the infected cells was performed to determine the subcellular localization of the Env protein expressed by FP*zenv*. The Env protein was expressed mainly in the cytoplasm (2a-2b-2c), and the intensity of the fluorescence signals was generally lower in cells infected with the recombinant than in the same cells infected with ZIKV (3b-3c). ZIKV did not infect the CEFs (3a). No immunofluorescence was detected in the FP-wild-type-infected cells used as negative controls (1a-1b-1c). **c** Expression of the *env* transcripts over time by FP*zenv* in replication-restrictive Vero cells. After infection of the Vero cells with FP*zenv*, the expression of the transgene was evaluated by RT-PCR every 3 days, over 27 days. The expression levels for FP*zenv* transcripts (661 bp) remained up to day 18 p.i.. Amplification of β-actin mRNA (518 bp) is shown. **d** Electron microscopy. Vero cells were infected with FP*zenv* to verify production of virus-like particles (VLPs). Left. Some empty VLPs were seen (white arrows), as well as clusters of FP*zenv* recombinants corresponding to the viral inoculum (black arrows) and DNA viral factories (V); bar, 0.2 µm. Inset, VLPs enlargement; bar, 50 nm. Right. ZIKV-infected cells (black arrows) were used as the positive control, and clusters of virus particles (50 nm, black arrows) were seen inside the cytoplasm; bar, 0.2 µm

Protein expression was investigated after infection of nonpermissive simian Vero cells with FP*zenv*, using Western blotting (Fig. 2**a**). A band of 54 kDa was always seen (Fig. 2**a**, lanes 4), which was also present when the Vero cells were infected with ZIKV (Fig. 2**a**, lanes 2) as the positive control, both when recognized by the mouse monoclonal antibody and the rabbit polyclonal antibodies. As expected, no specific band was present in the mock-infected cells (m, lanes 1) or in the cells infected with FP wild-type (wt, lanes 3).

### Env is expressed in the cytoplasm by FP*zenv*

**Fig. 3:**
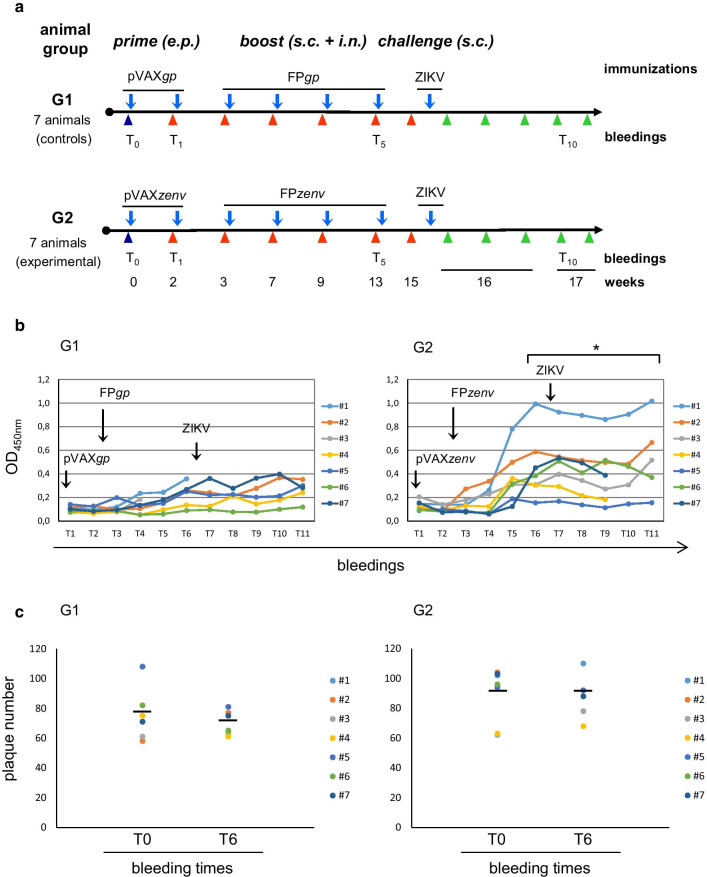
**a** Immunization protocols. Two different vaccination regimens (control, G1; experimental, G2) were followed using 7 mice per group. Two DNA recombinants were used for priming (pVAX*gp*, pVAX*zenv*, respectively), and two viral recombinants expressing the same genes were used for the boost (FP*gp*, FP*zenv*, respectively). The DNA*gp* and FP*gp* recombinants that contained the HIV-1 *gag/pro* genes were used as irrelevant immunogens. Each plasmid was administered in vivo by electroporation (10 + 50 µg/recombinant/mouse), and each virus was administered subcutaneously or intranasally (1 × 10^6^ PFU/recombinant/mouse). The challenge with ZIKV was administered subcutaneously at 1 × 10^5^ PFU/mouse. The mice were bled before each immunization, just before the ZIKV challenge (T6) and at further times after the challenge. **b** Analysis of the humoral immune response. The anti-Env antibody response was determined by ELISA, where Vero cells were infected with FPz*env* and then lysed, as the plate-bound antigen. Serum was obtained from all of the mice at different times before each immunization, as well as before and after the ZIKV challenge. Each line represents an individual animal. Total IgG ELISA titres are shown. An anti-ZIKV Env-specific binding antibody response was seen soon after vaccination (G2, T4). It can be noted that at 10 weeks postvaccination, after boosting the animals by the intranasal route, the antibody titer was significantly higher as compared to the control mice (G2 *vs* G1, T5; AUC, p < 0.05). OD_450_ is expressed after subtraction of the T0 values for each mouse. **c** Neutralizing activity using 1:50 serum dilution. Viral neutralization activity was determined using for each animal the pre-immune serum (T0) and sera from bleedings after the last immunization (T6). No inhibition of viral infectivity was found. Plaque numbers did not decrease when using hyper-immune or pre-immune sera (T6 *vs* T0) in the experimental *vs* the control animals (G2 *vs* G1)

To determine the subcellular localization of the Env protein expressed by FP*zenv*, the CEFs and Vero and MRC-5 cells were infected with FP*zenv* and analyzed by immunofluorescence (Fig. 2**b**). These data show that the Env protein was expressed mainly in the cytoplasm (Fig. 2**b**; 2a–c). The intensity of the fluorescence was similar to that observed in the same cells infected with ZIKV (Fig. 2**b**; 3b–c), except in the CEFs, which were not infected by ZIKV (Fig. 2**b**; 3a). The FPwt-infected cells were always negative, as expected (Fig. 2**b**; 1a–c).

### FP*zenv* expresses the transgene in Vero cells for more than 2 weeks

The expression of the *env* transgene after infection by FP*zenv* was also tested over time. The mRNA isolated from the infected Vero cells showed that the gene carried by FP*zenv* was amplified as a band of 661 bp, which was expressed for up to 18 days p.i. (Fig. 2**c**, lanes 2–7). The expression was similar up to 9 days p.i., and then gradually diminished from 12 to 18 days, and disappeared by 21 days p.i.. The negative control is represented by the mock-infected cells (Fig. 2**c**, T0, lane 1). β-actin RNA (518 bp) was similarly amplified in all of the samples, thus confirming the equal levels of total RNA across these different samples.

### Virus-like particles were found in Vero cells by electron microscopy

FP*zenv* was also used to infect the CEFs and MRC-5 and Vero cells for verification by electron microscopy of the formation of VLPs. Clusters of FP*zenv* recombinants were seen in the Vero cells corresponding to the viral inoculum (Fig. 2**d**, left, black arrows), as well as viroplasm (Fig. 2**d**, left, V) and a few empty VLPs (Fig. 2**d**, left, white arrows). The ZIKV-infected cells used as the positive control showed large viral progeny in the cytoplasm (Fig. 2**d**, right, black arrows). No VLPs were seen in the CEFs and MRC-5 cells infected with FP*zenv* (data not shown).

### Specific humoral immunity in mice primed with pVAX*zenv* and boosted with FP*zenv*

To develop a preventive vaccination strategy against ZIKV infection, an immunization protocol was set up to verify the capability of pVAX*zenv* and FP*zenv* recombinants to elicit antibodies against the Env protein, following a prime–boost strategy. The specific humoral responses were measured using ELISA, for individual sera samples from the immunized mice and the lysates of the FP*zenv*-infected cells as the plate-bound antigens (Fig. 3**b**). The anti-ZIKV Env-specific binding antibody response in experimental mice, which received the pVAX*zenv* plus FP*zenv* (Fig. 3**b**, G2), was evident soon after vaccination. In particular, the antibody titer became significantly higher at 10 weeks post-vaccination, as compared to the control mice immunized with the irrelevant pVAX*gp* plus FP*gp* recombinants (Fig. 3**b**, G2 *vs* G1, T5; p < 0.05). This increase corresponded to the T4 FP*zenv* boost, which was performed by the i.n. mucosal route. No significant specific immune responses were seen using the sera of the control mice (Fig. 3**b**, G1). No specific antibodies were seen with ELISA for plating of inactivated ZIKV or the recombinant domain-III ZIKV specific protein as a plate-bound antigen (data not shown).

### No neutralizing activity against ZIKV is seen

To determine the putative pre-challenge immune correlates of the protection against ZIKV, viral neutralization assays were performed using the sera at T0 and T6, for both the negative control (G1) and the experimental group (G2) (Fig. 3**c**). Inhibition of viral infectivity, expressed as a decrease in the number of lysis plaques after incubating the serum with the virus, was not detected. For each animal, plaque numbers did not decrease when using hyper-immune *vs* pre-immune sera (Fig. 3**c**, G2, T6 *vs* T0). Also, they did not essentially differ in the experimental (G2) and control (G1) animals. The number of plaques did not also change when different serum concentrations were used (data not shown).

### Challenge after dexamethasone-immunosuppression does not change the outcome of the mice

To determine the protective efficacy of the vaccine-induced immune responses, the mice were challenged with ZIKV after dexamethasone immunosuppression. Post-challenge sera from all of the animals of both groups were used to extract RNA, but no ZIKV genome was detected by RT-PCR (data not shown).

## Discussion

The link between ZIKV infections and severe congenital disease has prompted the development and evaluation of many candidate vaccines against ZIKV [1, 29]. These studies have been facilitated by prior experience with multiple successful flavivirus vaccine approaches, and by immunity evaluated in preclinical and clinical studies [29].

In particular, the antiparallel Env protein dimers found on virions are considered as the most suitable antigens for vaccine design, as this structural protein is the main target of neutralizing antibodies. The amino-acid sequence of the Env protein is also > 99% conserved across the three ZIKV lineages [56]. VLPs also share morphological and antigenic properties with infectious virus particles [57]. Thus, most vaccines have been developed to encode the ZIKV *PrME* sequence [22, 24], also using constructs with consensus *PrME* sequences from multiple ZIKV strains (downstream of the signal sequence of IgE) [17]. These vaccines have shown good safety profiles, induction of neutralizing antibodies, and protection from viremia. Inactivated vaccines have also been shown to be protective against virus challenges and to elicit neutralizing antibodies [16, 22, 25], although their development is no longer pursued. Live attenuated vaccines have also been investigated through the introduction of deletions or using chimeric flaviviruses [26, 58], and these have proved to be immunogenic and protective in mouse and nonhuman primate models [28]. In particular, the purified inactivated ZIKV by Larocca et al. [22] was modified by replacing the *PrME* signal sequence with the Kozak and the Japanese encephalitis virus leader sequence to optimize and enhance the *env* gene expression. The capsid-terminal 18 amino acid signal sequence of *PrME* [41, 42] was also used for the construction of a vaccinia-virus based recombinant [59]. This was obtained by the new Sementis Copenhagen Vector vaccine technology in CHO cells, and the deletion of the D13L gene, important for virus replication. Although homologous recombination was also used, clone selection by the fluorescent blue protein fused to the Zeocin resistance protein may be more cumbersome if compared to the clone selection with the *env*-specific radioactive probe that we always use.

Here we tested two novel recombinants expressing the *PrME* sequence in a prime-boost model under the capsid natural signal sequence, where pVAX*zenv* and FP*zenv* were used following the prime-boost strategy. To note, avipox-based recombinants do not cause the undesired side effects induced by vaccinia-based recombinants, and they are not neutralized in smallpox-vaccine experienced human subjects [33].

In spite of the progress that has been made, some issues still need to be resolved before licensing an effective prophylactic vaccine. In particular, pre-existing immunity to other flaviviruses can hamper the response to ZIKV immunization efficacy. It will also be important to verify a protective immune response against all the three ZIKV strains (i.e., West African, East African, Asian) [1].

With the aim being to improve immune responses against the *env* transgene, in the present study the mice were immunized following a heterologous prime–boost regimen. Furthermore, to determine whether a different administration route might improve the humoral responses, different routes were used for the FP*zenv* administration. In particular, dexamethasone-immunosuppressed BALB/c mice were used for the ZIKV challenge [54]. These data demonstrate that: (i) the ZIKV Env protein is correctly expressed by both human MRC-5 and simian Vero cells infected with FP*zenv*; (ii) FP*zenv* expresses the transgene in Vero cells for more than 2 weeks; and (iii) after mucosal administration of the avipox recombinant, the humoral response is significantly higher in the experimental mice, compared to the control mice.

The Western blotting shows that polyclonal and monoclonal antibodies can recognize the ZIKV Env protein expressed by FP*zenv*. The correct and long-lasting production of the transcript by Vero cells infected with FP*zenv* persisted for ≥ 18 days p.i., which, in spite of the mRNA decrease that started from day 12 p.i., might translate into long-lasting stimulation of the immune response. The intensity of the β-actin band was similar in the different samples, which supported the correct interpretation of the expression trend.

FP*zenv* was also tested for its production of VLPs using electron microscopy, and they were detected in low amounts in Vero cells. This suggests potential further stimulation of the immune system by these particles that mimic the original conformation of the virus.

A specific humoral response was obtained in the mice immunized with the experimental vaccine, with a significant increase when the FP*zenv* boost was performed by mucosal immunization. This suggests that in spite of the use of the conventional subcutaneous immunization route and the physiological injection of the virus by insect bite, a significant increase in the antibody response was obtained only when the mice were boosted intranasally. Most neutralizing antibodies target determinants in domain III or the fusogenic loop of domain II of the Env protein, and also protect after passive transfer in lethal mouse models of infection [60], which confirms the important role of humoral immunity. In our case, specific antibodies were not detected when using nonpurified ZIKV or domain III of the ZIKV Env protein. It is still not clear why the antibodies did not recognize nonpurified ZIKV, but we can hypothesize partial inaccessibility of the conformational epitopes on the mature virions. This might also explain the lack of binding to the DIII region of the E protein dimer that can recognize and neutralize ZIKV [61]. However, these antibodies did not neutralize ZIKV, as the number of viral plaques was almost the same when using the control and experimental sera, and was very similar to the number generated when the virus was incubated with preimmune serum (T0). It is known that, although many epitopes of ZIKV are very similar to those of other flaviviruses, higher concentrations of antibodies are needed for virus neutralization compared to other flaviviruses [29]. We have also previously demonstrated that the specific neutralizing response may be hidden by the aspecific activity, detected in naïve mice or in control mice immunized with irrelevant immunogens [44]. However, our previous study showed that vaccinated mice can be protected and survive also when a very low neutralization titer is used, with an increase in specific *vs* aspecific antibodies.

For the mice, weight losses after challenge progressed, although with no mortality among the animals, with some differences between the challenged control and experimental mice that did not reach significance. As all of the animals survived, it was also important to verify whether the virus was present in the serum soon after the challenge and in the 10 days thereafter. As the virus was never found, we can hypothesize inadequate immune suppression by dexamethasone, which might have translated into premature neutralization/inactivation of the virus, before its diffusion. This might be in agreement with the presence of mouse factors in the preimmune serum that can neutralize the virus.

Overall, although the mice were challenged s.c. to mimic the physiological virus inoculation, the data show that only the boost by the mucosal route enhanced the humoral responses. This might also suggest a future different use of the administration route with a possible reduction of the boosts to induce neutralizing antibodies.

Although virus neutralizing antibodies are generally considered as a surrogate of protective immunity for some licensed flavivirus vaccines [62], and different candidate vaccines exhibit neutralizing activity [28], results from different studies are using different not-comparable neutralization assays and repeated vaccine doses are required to have a titer able to protect the animals [29].

## Conclusion

Viral vectors represent a promising immunization strategy against emerging viruses, as they have already been shown to elicit both humoral and cell-mediated immunity. Their low production costs also contribute to favor their development. Different research groups have already used different strategies with vaccinia and adenovirus-based live vector candidates, to increase their efficacy in the absence of pathogenicity [16, 18, 22, 25, 28, 59]. Even if such recombinant vaccines can be highly immunogenic, questions remain as to their safety in some target populations [63]. Attenuated strains of vaccinia virus have also been tried, but antibodies against the vector were also found in the mouse model [64], which are not found by immunization with avipox-vector recombinants.

Susceptible mice models that maintain the natural competent immune responses should be the first choice to test vaccine efficacies, as impaired immune responses can hamper vaccine protection [65]. In our study, although BALB/c mice appear to be an adequate model for ZIKV infection, as it mimics the natural mild infection in human beings, with a short viremic phase [66], immune suppression by dexamethasone did not result in the expected viral replication in control mice. Therefore, different immune suppression strategies should be applied to reveal any protection before challenge as well as other animal models. Interferon-receptor-deficient immunocompromised mice can represent another strategy, by using AG129 Ifnαr1-/- mice, which lack receptors for both type I (α/β) and type II (γ) interferons [67], or mice treated with antibodies against interferon.

## Data Availability

The authors make the data supporting their findings available upon request to the corresponding or to the first author.

## Abbreviations

CEFs: Chicken embryo fibroblasts
DMEM: Dulbecco’s modified Eagle’s medium
FP: Fowlpox
VLPs: Virus like particles
ZIKV: Zika virus
